# Barriers to Puberty Talk between Mothers and Daughters: A Qualitative Study

**DOI:** 10.1055/s-0041-1729148

**Published:** 2021-06-28

**Authors:** Firoozeh Mirzaee, Malihe Pouredalati, Atefeh Ahmadi, Masumeh Ghazaznfarpour

**Affiliations:** 1 Nursing Research Center, Kerman University of Medical Sciences, Kerman, Iran; 2 Razi School of Nursing and Midwifery, Kerman University of Medical Sciences, Kerman, Iran

**Keywords:** content analysis, puberty, girls, mothers, qualitative study

## Abstract

**Objective** The aim of the present study is to explain the barriers to puberty talk between mothers and daughters.

**Methods** In the present study, the conventional content analysis method was used. The present study was conducted from September 2018 to August 2019 in Iran. The study population consisted of mothers and adolescent girls. The data was collected using purposeful sampling method. The sample consisted of 4 mothers and 6 girls that were interviewed using semistructured interviews. Data collection continued until data saturation was achieved. Data analysis was conducted as described by Graneheim et al. using NVivo 11 software.

**Results** In the present study, after exploring the views of the participants about barriers to puberty talk between mothers and daughters, one dominant theme emerged. Puberty talk is seen as an “inappropriate talk with a girl.” There were several subthemes, including “lack of mother's awareness regarding the school role, the busy schedule of the mother, and the adoption of alternatives to mother's talk with girls”.

**Conclusions** Different sociocultural factors affect puberty talk between mothers and adolescent girls. It is important that mothers and policy makers take these barriers into account.

## Introduction

The World Health Organization (WHO) defines adolescence as the period between childhood and adulthood in the age range between 10 and 19 years old. It marks the start of reproductive age. Adolescence is a period of life characterized with typical health, progressive, and authorized requests. Various dimensions of this period include biological processes, such as physical growth and weight gain, changes in body structure, and the development of the reproductive and sexual system. These typical descriptions are affected by socioemotional processes in different societies worldwide. 1 2 3

Psychological problems, infectious diseases, unsuccessful marriages, premature and risky pregnancies, injuries and deaths of mothers and children, as well as lasting physical and psychological problems can have a huge bearing in this period. 4 The fundamental health concerns caused by rapid changes during puberty are the source of the curiosity of adolescents. These concerns are related to physical, psychological, and mental changes, which highlight the psychosocial feature of sexual maturity. 5

Since adolescence is a time of vulnerability, obtaining health-related information from different sources is crucial to healthy choices made by adolescents. 6 There are three main sources of puberty information, including “mass media and their message about puberty,” “menstruation and related facts,” and “female-centered education through school and extracurricular programs, relevant books, and capacity building in the society by educating teachers and parents”. 7

The interaction of parents (as proximal social determinants of health) and adolescents, and the subjective norms/values of the parents and their role in the growth characteristics of adolescents can affect either positively or negatively their general health and welfare. 8 9 A study by Rembeck et al. 10 of the experiences of two girls, during adolescence exhibited that forging an active and close relationship with one's mother is essential in relieving stress and obstacles caused by the physical and sensitive changes in participants, which underscores the role of mothers as the first and most reliable source of support.

The results of a study performed in the USA showed that only 2% of teenagers gained their information from health staff. 11 Thus, available resources, convenience, sources of reference, educational meetings, and types of services can affect health practice and, therefore, improve or discourage health behaviors. 12 In girls going through menarche, mothers were the major source of information. Other sources, such as relatives (including an older sister) and teachers were scantly mentioned, and there were some regional disparities. In 41 studies, mothers were introduced as the source of information, with half of the girls reporting their mothers as the main source. 13 The Indonesia Demographic and Health Survey in 2012 reported that a quarter of the adolescent girls did not talk about menstruation with anyone before menarche, and that 17% of them were not aware that menstruation was a physical sign of puberty. 13

The study by Olfati et al. 14 in Iran showed that the knowledge the girls regarding puberty and their health attitudes and behaviors are significantly lower than expected due to the lack of proper information offered by parents to girls. The results of a study on Egyptian teenage girls exhibited that their level of knowledge, beliefs, and health presentation about puberty and menstruation was low. 15 Also, another study in Baghdad found that teenage girls were not sufficiently aware of puberty and menstruation, and this restrained their health practice due to their beliefs. 15

The points discussed above suggest that puberty talk between mothers and daughters is very important, but that this communication is affected by a variety of cultural, social, and economic factors, among others. However, some of these factors are still unknown. Despite multiple quantitative research and other studies conducted in Iran and in other countries, 16 17 18 some facets remain relatively unknown. In other words, people in different societies face various challenges. There are few resources available about barriers to puberty talk between mothers and daughters. Therefore, the present study was conducted to contribute to the literature in this field.

## Methods

The present study was conducted using qualitative and descriptive interviews with adolescent girls and mothers to explore their perspective on the meaning of puberty. This design offers a deeper insight into this issue, which is obscure and relatively unknown.

### Setting and Participants

The sample consisted of six girls and four mothers. They were recruited through verbal invitation, with a letter that explained the goals of the research. They were also assured of the confidentiality and anonymity of data reporting and management. The participants could withdraw at any moment during the process of data collection. Prior to the study, an informed consent form was obtained from the participants. The inclusion criteria were: speaking Farsi, experience of puberty, and having a daughter who has experienced puberty. Reluctance to continue the interview was excluded from the present study. A total of 6 women and 11 girls were invited to participate in the study; 2 women refused to participate in the survey due to time restrictions, and 1 girl withdrew from the study due to discomfort with an interview.

### Data Collection

The interviews were conducted by an M.P, who is a midwife and expert in the subject. She collected data using face-to-face interviews with the participants. The present study was conducted from November 2018 to July 2019. For this purpose, a semistructured questionnaire was designed. The interviews with the girls began with an open-ended question about how the girls experienced puberty. Some of interview questions included: “Would you like to explain puberty to me?” or “Which factors do affect puberty talk between your mother/ girl?” There were some probe questions used during interviews, such as: “Can you elaborate more on this subject?” or “Can you give me an example for this issue?” All participants were interviewed at school, in a private room. Individual interviews lasted between 30 and 45 minutes. All interviews were voice-recorded using an MP3 recorder (model ICD-PX470, Sony electronic inc. Made in China), and then were transcribed verbatim. The interviews were recorded in Persian language and then translated into English. The participants were selected from among diverse individuals. Data collection was sustained until saturation was achieved. that is, after 8 interviews. The last two interviews did not add any extra information.

### Data Analysis

Data collection and analysis were conducted simultaneously. The conventional content analysis method was used for data analysis, as described by Graneheim et al. 19 The interviews were reread and relistened to reinforce deeper engagement with the data. Codes were involved to small parts of the transcripts using NVivo 11. Emerging themes were identified and checked to determine their relevance regarding the data. Initial themes were refined and divided into subthemes by reviewing the data to establish clear patterns. Quotes that explained the themes were assigned a code. A total of 1,235 codes was achieved after merging 3 subthemes, and 1 theme was obtained.

### Rigor and Reflectivity

In the present study, we used several methods to ensure methodological rigor. All researchers were conversant with research methods and expert in the field of midwifery and puberty-related matters. Field notes were obtained from each interview (data triangulation). Throughout the study, we reflected on the analytic process (the investigator's triangulation). We also held meetings with the research team to discuss scientific and organizational aspects of the study (peer debriefing). The writing of the present article was guided by the combined criteria for reporting qualitative research (COREQ).

## Results

The study participants comprised 10 mothers and girls (4 mothers with adolescent girls at the age of puberty and 6 girls). The mothers were in the age range between 39 and 51 years old, and the girls were between 13 and 15 years old. The demographic characteristics of the participants are presented in Table 1 . Quotes are shown in italics. The statements belonging to mothers and girls are marked with M and G, respectively. In exploring the views of the participants about the barriers to puberty talk between mothers and girls, one dominant theme emerged: “Inappropriate relationship with girls.”

**Table 1:** Characteristics of the Participants *

Characteristics	Value
Mothers	
Employment status	
Employed	2(50)
Housewife	2(50)
Race	
Iranian	4(100)
Number of children	
> 2	2(50)
≥ 2	2(50)
Income status	
> $45	1(25)
$45–100	2(50)
≥ $100	1(25)
Civil status	
Married	4(100)
Level of education	
Diploma	1(25)
B.A degrees	2(50)
M.A degrees	1(25)
Experience of raising an older daughter	
Yes	2(50)
No	2(50)
**Girls**	
puberty experiences, y	
> 3	2(33.33)
≥ 3	4(66.67)
Nationality	
Iranian	6(100)

* Values are expressed as N (%).

Other subthemes included “lack of mother's awareness regarding the role of schools, busy schedule of mothers, and replacement of communication with mother.”

### Inappropriate Relationship with the Girl

In the present study, mothers and girls admitted that one of the main barriers to the puberty talk between mothers and daughters was an inappropriate relationship. This dominant theme comprised three subthemes, which are described below ( Chart 1 ).

**Chart 1:** “Inappropriate relationship with girl” as a dominant theme in barriers to puberty talk between mothers and daughters

Theme	Subtheme	Category
Inappropriate relationship with girls	Mother's unawareness of the role of the school	Mother's lack of awareness regarding the role of school health educators.
		Delegating maternal duties to the school's health educators.
	Busy schedule of the mother	Employed mothers
		Mother's responsibility to take care of other children
	Using alternatives to mother's talk with girls	Communication with peers
		Communication with an older sister
		Communication on the Internet
		Generational gap and disparity of views

### Lack of Mother's Awareness Regarding the Role of the School

Lack of mother's awareness regarding the role of the school was one of the main barriers to appropriate puberty talk between mothers and daughters. This category covered two issues: “lack of mother's awareness regarding the role of the school's health counselors and delegation of maternal duties to the school's health educators.”

In the view of mothers, schools provide complete information about puberty to their daughters, and they do not need to discuss these issues with their daughters. In other words, they delegated their maternal job to the school. This overreliance on the school undermined the interaction of mothers and adolescent daughters.

The statement by one of the mothers (M3, 41 years old, employed) lends credit to this point: “I think the school offers girls all the necessary information about puberty and, hence, I do not need to talk to my daughter about it.”

### Mothers with a Busy Schedule

According to the participants, one barrier to this mother-daughter communication was the busy schedule of the mothers. “Employed mother and the responsibility of taking care of other children” fall into this category. In the case of employed mothers, they are too busy to talk about puberty with their daughters due to their occupational concerns. When there are many children and mothers must take care of other children, they also do not have enough time to talk with their daughters about puberty.

For example, one of the girls (G2, 14 years old, 4 years of puberty experience) asserted, “…my mother does not have time to talk about this sort of things to me. She is employed and, when coming back from work, she is too tired to talk.” On the other hand, a mother (M2, 49 years old, housewife) stated: “I have two other children to look after, and this leaves me no time to talk about puberty with my daughter.”

### Using Alternatives to Mother's Talk

“Generation gap in terms of views, talking with peers or an older sister, and online communications” are other categories of this subtheme.

According to the participants, the generation gap between mothers and daughters is the cause of divergent beliefs between the two generations. As a result, they prefer to discuss these puberty issues with their peers as a way of substituting the relationship with their mothers.

As stated by one of the girls (G4, 15 years old, 6 years of puberty experience), “I can easily talk about puberty with my peers because we understand each other.”

Intellectual differences between generations present another barrier to proper puberty talk between mothers and girls.

However, there are disparity in values, beliefs, and lifestyles due to the development of the society.

This divergent lifestyle is viewed as a barrier to puberty talk between mothers and daughters.

This point is raised by one of the girls (G6, 15 years old, 5 years of puberty experience): “Due to the intellectual and generational difference between my mother and I, we have trouble understanding each other, since we have different values and attitudes.”

Mothers who had older girls suggested that their adolescent girls preferred to talk with their older sisters because the older sister could resonate with their experience more effectively. Girls feel less ashamed and embarrassed to talk with their sister rather than with their mother.

This point was also underscored by another girl (G1, 13 years old, 2 years of puberty experience): “I have an older sister and I ask her most of my puberty questions, because I'm ashamed of talking to my mother about these things.”

Girls' access to the cyberspace is another barrier to effective talk between mothers and their daughters. The girls can easily obtain necessary information about puberty from the Internet.

In this regard, one of mothers (M1, 51 years old, housewife) mentioned: “Today, children have access to the Internet, and my daughter gets all the information she needs from the Internet, so she talks less about these issues with me.”

## Discussion

All family members are involved in teaching puberty subjects to adolescents, but the role of the mother is more significant than that of others, as adolescent girls often learn healthy behaviors from their mothers. 20 21 22 Some studies have also reported that parents are the key sources of information about reproductive health for their children. According to the study by Nwagwu in Nigeria, the major sources of information about reproductive health of adolescent girls are parents (56.1%), friends (53.18%), books (45.56%), teachers (44.15%), the Internet (45.19%), and health centers (54.14%). 23

Many studies in Iran and in other parts of the world have shown that peers and the media are the primary sources of information about the reproductive health of adolescent, 24 25 which is at odds with evidence from India, Nagpur, Tigray, and Nigeria, where mothers are the major source of information. 26 27 28

In our study, communication through cyberspace and peers were major sources of information, which is consistent with the aforementioned studies. We found that these forms of communication substituted puberty talk between mothers and daughters. Kumar et al. 29 demonstrated that mothers and sisters are the main source of information about puberty for 75 and 8.64% of girls, respectively. The findings of our study revealed that girls preferred talking to their older sister, which is aligned with the aforementioned results.

Mothers can answer many of the questions of the girls regarding reproductive subjects, which can improve the quality of the mother-daughter relationship and mitigate the load of physical, psychological, and social problems and unhealthy behaviors associated with adolescence. 30 It seems that mothers are the best reference to provide basic information about reproductive health to their daughters, but the sense of embarrassment, insufficient knowledge, superstitions, and misunderstandings of mothers about reproductive health can deter them from imparting their knowledge to the adolescents. 31 Mothers need to be sufficiently aware of the physical and emotional changes of their teenagers. 32

In the study by Shahhosseini et al., 33 healthcare providers highlighted the role of mothers as the most reliable source of information for adolescents. From the perspective of mothers, describing menstruation to adolescent girls is their duty, but they pointed to the difficulty of explaining menstruation to the girls. 34 Contrary to these findings, our results suggested that the lack of awareness by part of the mothers regarding the role of school health educators and delegating maternal duties to school health educators deter them from getting involved in in puberty education of their daughters. In line with our findings, another study has shown that students are not happy about receiving sexual education from their families. 24

In the study by Kamalikhah et al., 24 parents were weighed barrier to discussions about sexual reproductive health. Talking about sexual health issues with adolescents was associated with a sense of embarrassment in some mothers. 35 As a result, the relationship between parents and adolescents should be fostered through cooperative training programs. Some studies have stressed the effects of formal education on health issues of female students. 35

An inappropriate mother-teenager relationship can hamper a teenage girl's access to the most vital sources of information, paving the way for flawed sources of information, which can provoke a plethora of health problems.

Our results suggested that employed mothers usually do not have enough time to talk with girls about puberty, since they spend most of their time at work. Also, mothers who take care of other children at home lack time to talk about puberty with their daughters.

Unlike the results of our study, the results of a study in Nigeria revealed a significant statistical association between the employment of mothers and the practice of good menstrual hygiene. 36

The findings of our study have shown that the generation gap between mothers and daughters is also a barrier to proper communication between mothers and girls. This intellectual distance may lead girls to seek guidance from friends or sources other than their mothers regarding puberty issues.

Therefore, it is necessary that health professionals adopt an approach that stresses training mothers and informing girls about puberty. These training programs for mothers should address how to forge a close mother-daughter relationship and how to remove barriers to the discussion of puberty or of the health consequences that originate from the lack of knowledge.

## Conclusion

The present study was conducted in Iran, so the findings might have limited generalizability. However, this is an essential characteristic of all qualitative studies. However, the results of the present study could be generalized to societies with the same cultural backgrounds. Another limitation of the present study is the lack of interviews with counseling teachers who advise girls in schools. It is suggested that they be interviewed in future studies.
